# Fiber-Optic System for Dual-Modality Imaging of Glucose Probes ^18^F-FDG and 6-NBDG in Atherosclerotic Plaques

**DOI:** 10.1371/journal.pone.0108108

**Published:** 2014-09-18

**Authors:** Raiyan T. Zaman, Hisanori Kosuge, Guillem Pratx, Colin Carpenter, Lei Xing, Michael V. McConnell

**Affiliations:** 1 Division of Cardiovascular Medicine, Stanford University School of Medicine, Stanford, California, United States of America; 2 Division of Radiation Physics, Stanford University School of Medicine, Stanford, California, United States of America; Brigham and Women’s Hospital, Harvard Medical School, United States of America

## Abstract

**Background:**

Atherosclerosis is a progressive inflammatory condition that underlies coronary artery disease (CAD)–the leading cause of death in the United States. Thus, the ultimate goal of this research is to advance our understanding of human CAD by improving the characterization of metabolically active vulnerable plaques within the coronary arteries using a novel catheter-based imaging system. The aims of this study include (1) developing a novel fiber-optic imaging system with a scintillator to detect both ^18^F and fluorescent glucose probes, and (2) validating the system on *ex vivo* murine plaques.

**Methods:**

A novel design implements a flexible fiber-optic catheter consisting of both a radio-luminescence and a fluorescence imaging system to detect radionuclide ^18^F-fluorodeoxyglucose (^18^F-FDG) and the fluorescent analog 6-(N-(7-Nitrobenz-2-oxa-1,3-diazol-4-yl)amino)-6-Deoxyglucose (6-NBDG), respectively. Murine macrophage-rich atherosclerotic carotid plaques were imaged *ex vivo* after intravenous delivery of ^18^F-FDG or 6-NBDG. Confirmatory optical imaging by IVIS-200 and autoradiography were also performed.

**Results:**

Our fiber-optic imaging system successfully visualized both ^18^F-FDG and 6-NBDG probes in atherosclerotic plaques. For ^18^F-FDG, the ligated left carotid arteries (LCs) exhibited 4.9-fold higher radioluminescence signal intensity compared to the non-ligated right carotid arteries (RCs) (2.6×10^4^±1.4×10^3^ vs. 5.4×10^3^±1.3×10^3^ A.U., P = 0.008). Similarly, for 6-NBDG, the ligated LCs emitted 4.3-fold brighter fluorescent signals than the control RCs (1.6×10^2^±2.7×10^1^ vs. 3.8×10^1^±5.9 A.U., P = 0.002). The higher uptake of both ^18^F-FDG and 6-NBDG in ligated LCs were confirmed with the IVIS-200 system. Autoradiography further verified the higher uptake of ^18^F-FDG by the LCs.

**Conclusions:**

This novel fiber-optic imaging system was sensitive to both radionuclide and fluorescent glucose probes taken up by murine atherosclerotic plaques. In addition, 6-NBDG is a promising novel fluorescent probe for detecting macrophage-rich atherosclerotic plaques.

## Introduction

According to the World Health Organization, approximately one third of all deaths in developed countries are related to coronary artery disease (CAD), characterized by the buildup and disruption of atherosclerotic plaques within the coronary wall [1]. Atherosclerosis is a progressive inflammatory condition that underlies CAD–the leading cause of death in the United States [2]. A subset of plaques is thought to be unstable and prone to rupture (“vulnerable”), which can cause life-threatening acute coronary syndromes. The most common type of vulnerable plaque is an inflamed thin-cap fibroatheroma (TCFA), which is thought to account for 60% to 70% of coronary events [3]. If we are able to identify these vulnerable plaques, we may be able to guide treatment to reduce the risk of heart attack and the debilitating aftermath, reducing the burden on individuals as well as society.

The current clinical paradigm for detecting CAD is angiography, which only evaluates the luminal encroachment of the disease, without providing information about plaque extent and content [4]. There is a wide range of molecular imaging modalities employed for detection and characterization of atherosclerosis, primarily in large vessels. Examples include positron emission tomography (PET) with ^18^F-FDG [5], magnetic resonance imaging (MRI) using ultra-small super-paramagnetic iron oxide (USPIO) probes [6], [7], single photon emission computed tomography (SPECT) using VCAM-1-specific ^99m^Tc-labeled peptidic sequences [8], and the iodinated nanoparticulate contrast agent N1177 for computed tomography (CT) [9]. However, application of these external imaging approaches to coronary atherosclerosis has been challenging due to small coronary size, cardiac motion, and detecting adequate signal and contrast. Recently, an intravascular near infrared fluorescence (NIRF) approach employing a protease-activatable NIRF agent has been applied to image plaque inflammation in the rabbit aorta [10].

Several studies have shown that ^18^F-FDG can be a marker of metabolically active (“vulnerable”) plaques due to its uptake by inflammatory macrophages in the carotids and aorta [11]–[13]. ^18^F-FDG has the major advantage of being FDA approved for cardiac and cancer imaging; thus, clinical transition may be more easily achieved. However, PET ^18^F-FDG detection in coronary plaque is still challenging due to the small size of such plaques, signal blurring due to motion, and obscuring FDG uptake by adjacent myocardium [14]. An intravascular molecular imaging approach [15] has the potential to overcome these limitations.

As fluorescent probes may provide higher intravascular signal [16] than radionuclides without the need for ionizing radiation, they also merit study. Thus, to complement the study of ^18^F-FDG, we have selected 6-(N-(7-Nitrobenz-2-oxa-1,3-diazol-4-yl)amino)-6-Deoxyglucose (6-NBDG), a fluorescent glucose analog. It has higher signal than 2-NBDG [17] and does not degrade to non-fluorescent products due to its inability to be phosphorylated by hexokinase [18]. To the best of our knowledge, 6-NBDG has not been studied for detecting inflammation in atherosclerosis. We developed the dual-imaging system as the ideal method to compare radionuclide and fluorescent agents *in vivo.*

Macrophage infiltration in atherosclerotic plaques plays a vital role in the progression of atherosclerosis [19]. As a result, macrophages have become widely recognized as a key target for atherosclerosis imaging, since they contribute significantly to the progression of atherosclerosis [20]. Thus, in this feasibility study we implement a novel dual-modality fiber-optic system to detect atherosclerotic plaque by targeting macrophages in inflamed metabolically active plaques using ^18^F-FDG and 6-NBDG. Therefore, the aims of this study were to (1) develop this fiber-optic imaging system with a scintillator to detect ^18^F and fluorescent glucose probes, and (2) validate the system on *ex vivo* macrophage-rich murine plaques with confirmatory external optical imaging and autoradiography.

## Materials and Methods

### 2.1. Metabolic imaging with glucose probes

Two glucose analogs were studied–^18^F-FDG and 6-NBDG. ^18^F-FDG is a radionuclide consisting of the positron-emitting radioactive isotope fluorine-18 instead of hydroxyl group (OH) at the 2′ position of the glucose molecule (MW 181.15 g/mol). The 2′ hydroxyl group (OH) in normal glucose is needed for further glycolysis; thus, ^18^F-FDG cannot be further metabolized and is retained in macrophages in atherosclerotic plaques [11].

The 6-NBDG molecule consists of the small NBD fluorochrome, a nitrobenzoxydiazoamino group (MW 342.26 g/mol) attached to D-glucose at the C-6 position and fluoresces at 540 nm when excited at 465 nm (Invitrogen, Life Technologies, Grand Island, NY USA). This fluorescent probe has been shown to be transported into the cell by the same glucose transporters (GLUT) as glucose, but does not undergo phosphorylation by hexokinase (unlike ^18^F-FDG) due to the NBD fluorochrome and never enters the glycolytic pathway, a reaction that causes 6-NBDG to accumulate within the cell [18], [21].

### 2.2. System setup

#### 2.2.1. Catheter radionuclide imaging (CRI) system

**Figure 1:**
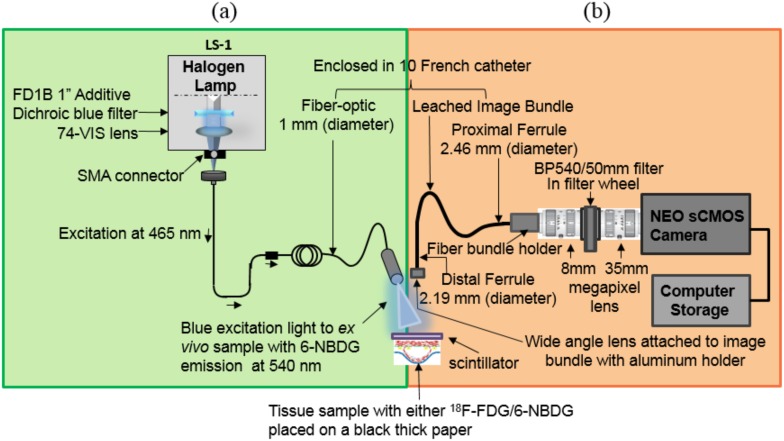
Schematic diagram of the dual-modality fiber-optic imaging system. The catheter fluorescence imaging (CFI) system combines components shown in (a) and (b) including a 540 nm filter placed in a filter wheel to detect glucose probes such as 6-NBDG fluorophore. Scintillating screen is removed during the fluorescence measurement. The catheter radionuclide imaging (CRI) system is shown in (b) to detect ^18^F-FDG radionuclide when the CFI light is turned off, no emission filter is in the optical path, and a scintillating screen is used in front of the wide-angle lens. The novel system was placed inside a light-tight black box to prevent any ambient light.

The CRI system (Figure 1) consists of a wide-angle lens (4.9 mm×3.6 mm), providing 180 degree view of an artery, with a focal length of 3.85 mm (Dowell Electronic Technology Co. Ltd, Minzhi Longhua Shenzhen, Guangdong, China), attached to the distal ferrule (diameter 2.19 mm) of a leached image fiber bundle (Schott North America, Inc. Southbridge, MA USA). The proximal end (diameter 2.46 mm) of the fiber bundle is connected to an 8 mm megapixel fixed-focal-length lens and the other end of this lens is attached to a filter wheel. The leached image bundle runs parallel to the excitation fiber of the CFI and is enclosed within a 10-French catheter. A 35 mm lens connects the filter wheel to a CMOS camera (NEO sCMOS, Andor Technology, CT, USA), and a computer.

The novelty of the CRI system is the use of an inorganic scintillating screen [22] (10×10 mm with 0.5 mm thickness, MTI Corporation. Richmond, CA, USA) of Lutetium Oxyorthosilicate (LSO), placed in the front of the wide angle lens to convert the β-particles of the positron emission signal into visible light due to radioactive decay. The efficiency of LSO is 24,000 photons/MeV and emits primarily in green with an emission peak at 420 nm. This light was then captured with the camera using deep thermoelectric cooling at −40°C for minimizing the background signal from the temperature-dependent dark current, hot pixel blemishes, and vibration. The exposure time is set to 10 seconds, which enabled sufficient signal from the *ex vivo* plaques. Both the CRI and CFI system were placed in a light-tight black box to prevent ambient light.

#### 2.2.2. Catheter fluorescence imaging (CFI) system

The CFI system (Figure 1) requires several additional components compared to the CRI system. These include a white LS-1 tungsten halogen light source (Ocean Optics, Dunedin, FL, USA), then a 1″ dichroic blue color low pass filter with transmission between 390 and 480 nm with cutoff at 505±15 nm wavelength (FD1B Thorlabs, Inc. New Jersey, USA) is placed in the built-in slot of the LS-1 to transmit only blue light through the excitation fiber (core diameter 1 mm) and deliver it to the sample at a 45 degree angle. The resulting photo-stimulated green light emitted at 540 nm wavelength from the 6-NBDG-enriched sample is collected with the wide-angle lens and a bandpass emission filter BP540/50 mm (filter BP 540 nm×10 nm OD4 50 mm, Edmond Optics, USA) placed inside the filter wheel. Then, the emission light is captured by the shared wide-angle lens and the CMOS camera cooled at −40°C. The exposure time for the fluorescence imaging was also set to 10 seconds, as with the CRI system.

#### 2.2.3. External optical imaging system (IVIS-200)

The IVIS-200 system (Xenogen Corporation/Caliper Life Sciences, Alameda, CA, USA) provides for bioluminescence and fluorescence optical-based imaging and was used to validate the CRI/CFI system. Samples were placed inside the light-tight imaging chamber and the field-of-view set to 1.5 cm for both 6-NBDG and ^18^F-FDG samples, with the highly sensitive charge-coupled device (CCD) camera system cooled to −90°C. For both 6-NBDG and ^18^F-FDG imaging the binning factor was set at medium (8×8 pixels). For 6-NBDG fluorescence imaging, Green Fluorescent Protein (GFP) (excitation: 445–490 nm; emission: 515–575 nm) and GFP-background (410–440 nm) filters were used to obtain the correct excitation and emission signal from the 6-NBDG-enriched samples. The fluorescence images were collected over 0.5–10 seconds and 0.5–30 seconds for *in vitro* and *ex vivo* imaging, respectively. During ^18^F-FDG radionuclide imaging, a scintillator screen was placed on top of the tissue sample and the bioluminescence mode was used to image the radioluminescence from the β-particles of the positron emission signal from ^18^F-FDG radioactive decay.

#### 2.2.4. Autoradiography

Autoradiography was used to directly image the radiation emitted from the ^18^F-FDG tissue samples with high spatial resolution [23] as further validation. Samples were placed in contact with a super resolution storage-phosphor screen (12.5×25.2 cm, PerkinElmer Inc., MA, USA). Over a sufficient exposure time (48 hours), emitted beta particles were recorded as an image on the storage-phosphor screen. The film was then read out by a Cyclone reader using a spatial resolution of 600 dpi (PerkinElmer Inc., Waltham, MA, USA). Using these techniques, the spatial distribution of radioisotope uptake of the tissue sample was recorded [24].

### 2.3. Experimental procedures

#### 2.3.1. *In vitro* validation of 6-NBDG macrophage uptake

To establish 6-NBDG fluorophore as a potential molecular marker for detecting macrophage-rich atherosclerotic plaque, we studied macrophage uptake *in vitro* prior to *in vivo* studies. See Methods S1 and Results S1 for supplementary information on 6-NBDG uptake by macrophages.

#### 2.3.2. *Ex vivo* imaging of ^18^F-FDG/6-NBDG uptake

*Ex vivo* experiments were conducted according to protocol approved by the Administrative Panel on Laboratory Animal Care (APLAC permit number 9941) of Stanford University. All surgery was performed under anesthesia (2% Isoflurane), and all efforts were made to minimize suffering. Macrophage-rich atherosclerotic lesions were created in the left carotid arteries (LCs) of diabetic, hyperlipidemic FVB/NJ mice, as studied extensively by our laboratory [25]–[28]. In brief, 8-week-old male mice (n = 6) were fed a high-fat diet containing 40% kcal fat, 1.25% (by weight) cholesterol and 0.5% (by weight) sodium cholate (D12109, Research Diets, Inc., New Brunswick, NJ, USA). After 1 month on the diet, diabetes was induced by 5 daily intra-peritoneal injections of streptozotocin (STZ, 40 mg/kg, Sigma-Aldrich). Two weeks after the initiation of diabetes, the left common carotid artery was ligated below the bifurcation with the use of 5-0 silk ligature (Ethicon) while the mice were anesthetized with 2% Isoflurane. The non-ligated right carotid serves as an internal negative control. Two weeks after carotid ligation, mice were injected intravenously (IV) with either ^18^F-FDG (0.964–1.437 mCi) or 6-NBDG (1 mg/mL concentration of 500 µM, 177 µL in volume) after 6 hours of fasting (n = 3/group). One hour after injection, ligated LCs and non-ligated right carotid arteries (RCs) were harvested and cut open longitudinally and placed individually on black paper for *ex vivo* imaging, also with the heart sectioned as a positive control. Samples from mice injected with ^18^F-FDG or 6-NBDG were imaged *ex vivo* with the fiber-optic imaging system. The scintillating screen was placed in contact with the ^18^F-FDG-enriched tissue sample to capture all light converted from the β-particles of the emission signal of ^18^F-FDG decay. The best image quality can be achieved when the scintillator is in contact with the tissue or ≤100 µm air gap, so energy can be deposited in the scintillator by all the β-particles. For 6-NBDG, imaging was performed with the scintillating screen removed. The exposure time was chosen to be 10 seconds based on prior experiments to achieve adequate signal intensity. To validate these findings, LCs and RCs, plus the heart, were also imaged using the IVIS-200 system (see 2.2.3 for system setup).

For radionuclide imaging with both the fiber-optic and IVIS-200 systems, each sample was placed under the scintillator screen to convert β-particles of the emission signal into visible light due to radionuclide decay of ^18^F-FDG. For the fiber-optic system, each sample (LC, RC, and heart) was imaged separately due to the small field-of-view (5 mm).

**Figure 2:**
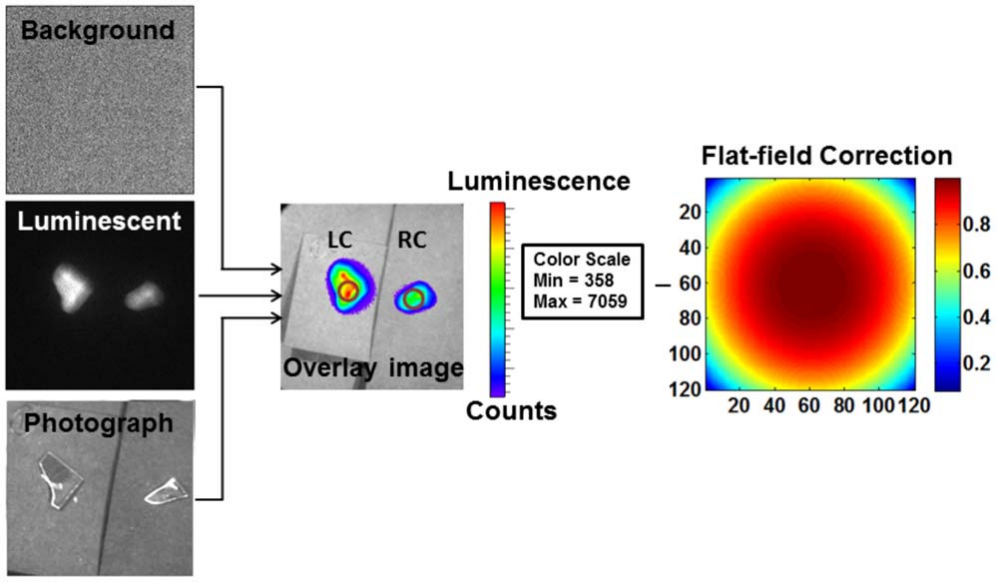
ROI selection on IVIS-200 images. Three images–background, luminescent, and photograph were collected using IVIS-200 to create an overlay image. Then, a region of interest (ROI, shown with circle) was selected on the left carotid (LC) and right carotid (RC) arteries. The overlay image was corrected for field flatness, which was specific to the camera used in the IVIS-200 imaging system.

Both radioluminescence and fluorescence signals from the LCs and RCs were calculated based on the selected ROIs (same size for all samples). An average of mean signal intensity was then calculated in arbitrary units (A.U.) based on the CRI and CFI images. An average radiance was calculated based on images of ^18^F-FDG enriched tissue by correcting for field flatness specific to the camera used in the IVIS-200 imaging system (Figure 2). For the fluorescence study, an average of mean radiant efficiency was calculated after a CF was applied to the IVIS-200 images of 6-NBDG enriched tissue.

#### 2.3.3. Autoradiography of *ex vivo* tissue with ^18^F-FDG

LCs and RCs were placed side by side in contact with a storage-phosphor screen over 48 hours, with the heart placed at a distance to avoid overlap of the radioisotope decay due to the high emission from heart tissue.

### 2.4 Statistical analysis

A pairwise two-sample Student’s t-test was performed to compare *ex vivo* signal intensity from ligated LCs and non-ligated RCs for both the ^18^F-FDG and 6-NBDG experiments. The underlying distribution was found to be normally distributed according to QQ-plots. As all animals had similar weight and dose, these factors were not considered in the statistical analysis. *Ex vivo* analyses were performed using MATLAB software.

We used P<0.05 for statistical significance for all *in vitro* and *ex vivo* analyses.

## Results

### 3.1. ^18^F-FDG imaging

**Figure 3:**
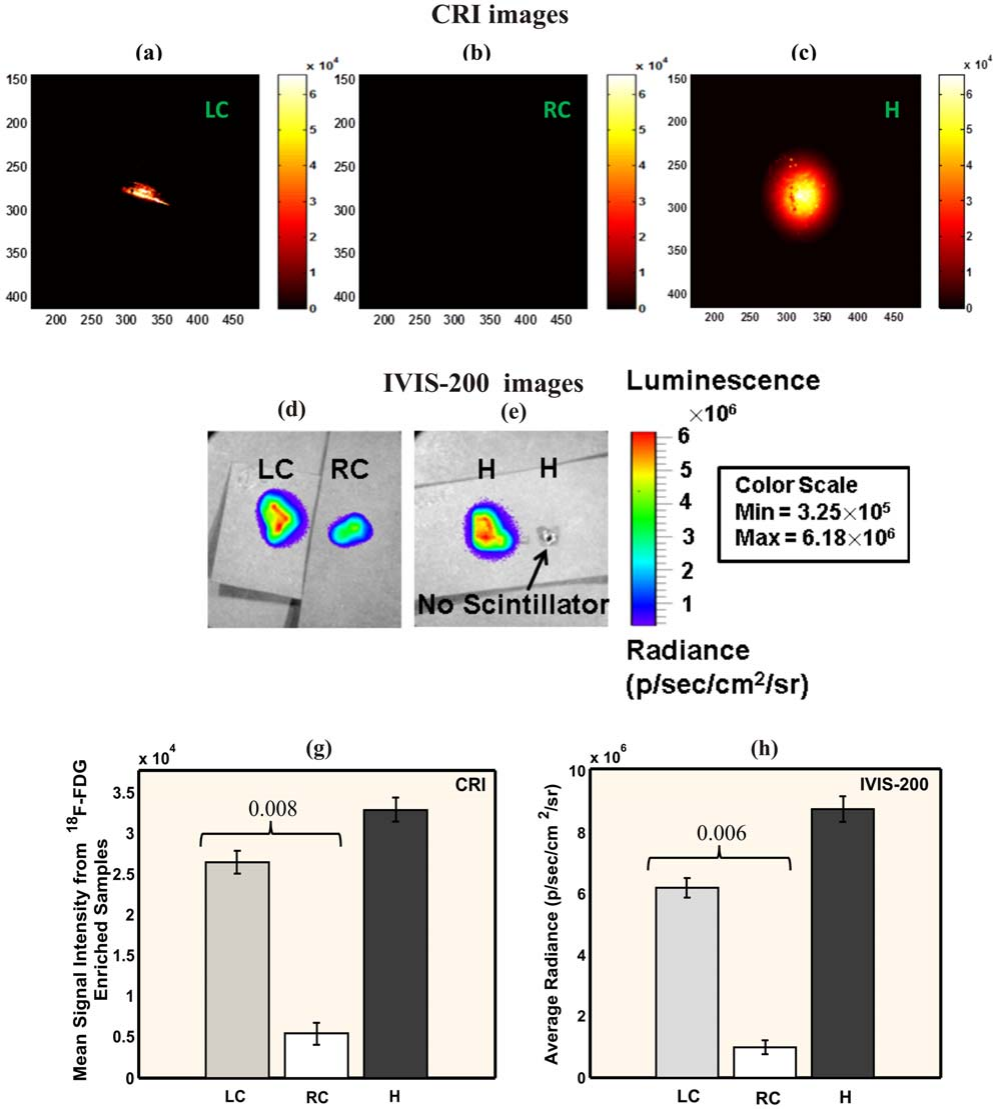
Radioluminescence imaging of *ex vivo* murine carotid arteries. Radioluminescence imaging was taken one hour after ^18^F-FDG intravenuous injection (0.964 mCi) with the CRI system: (a–c) and with the IVIS-200 system: (d–e). (a) LC: ligated left carotid artery, (b) RC: non-ligated right carotid artery artery (negative control), (c) H: heart (positive control). (d) both LC and RC artery under scintillator, (e) left: heart under scintillator, right: heart without scintillator; (g) average signal intensity of tissue samples from the CRI system images (h) average radiance of the samples from the IVIS-200 system images. High radioluminescence signal from LCs was detected by the CRI system and confirmed by IVIS-200.

**Figure 4:**
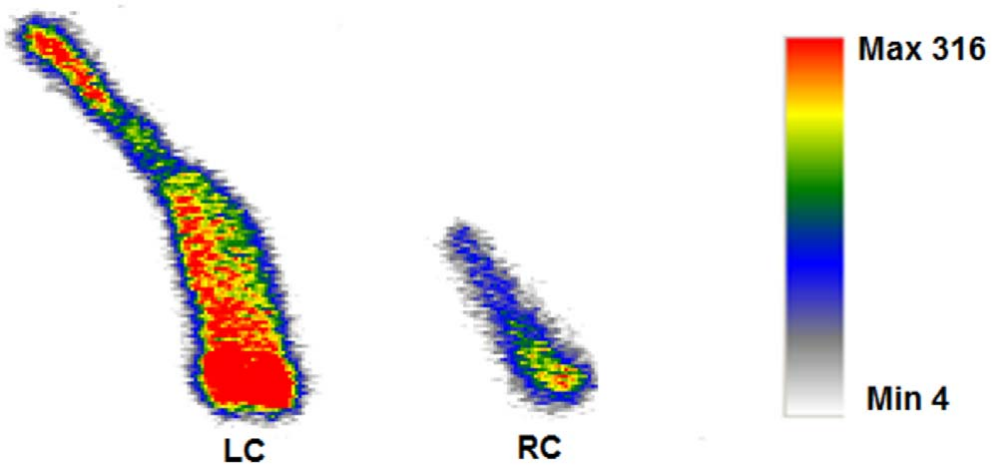
Autoradiography imaging for confirmatory purpose. *Ex vivo* autoradiography from a mouse injected with ^18^F-FDG. The ligated left carotid artery (LC) showed high amount of ^18^F-FDG uptake compared to the non-ligated right carotid artery (RC).

*Ex vivo* radio-luminescence images (Figures 3a–c) with the CRI system detected a 4.9-fold higher radioluminescence signal emission from the ligated LCs post-^18^F-FDG compared to the control RCs (2.6×10^4^±1.4×10^3^ vs. 5.4×10^3^±1.3×10^3^ A.U., P = 0.008; Figure 3g), with high ^18^F-FDG signal also detected in the heart. Radioluminescence imaging with IVIS-200 (3d-e and h) confirmed the high signal intensity in the LCs compared to RCs (6.18×10^6^±3.25×10^5^ vs. 9.88×10^5^±2.25×10^5^, p/sec/cm2/sr, p = 0.005) and in the heart (8.75×10^6^±4.25×10^5^). For further confirmation, autoradiography was performed on the *ex vivo* carotids (Figure 4), with high ^18^F-FDG signal observed in the LCs vs. RCs. All mice were imaged at the same day to avoid system variability, so the dose of ^18^F-FDG was adjusted (0.964–1.437 mCi) for each mouse to account for decay over sequential IV injections of ^18^F-FDG. This sequential dosing of ^18^F-FDG was performed as absorbed dose in organ depends on the radionuclide, the amount of activity, geo-metrical distributions of the radionuclide and on the residence time in the tissue [29], [30]. Thus, the mouse imaged last received the largest ^18^F-FDG dose so the radioluminescence signal would be comparable with the first animal.

### 3.2. 6-NBDG imaging

**Figure 5:**
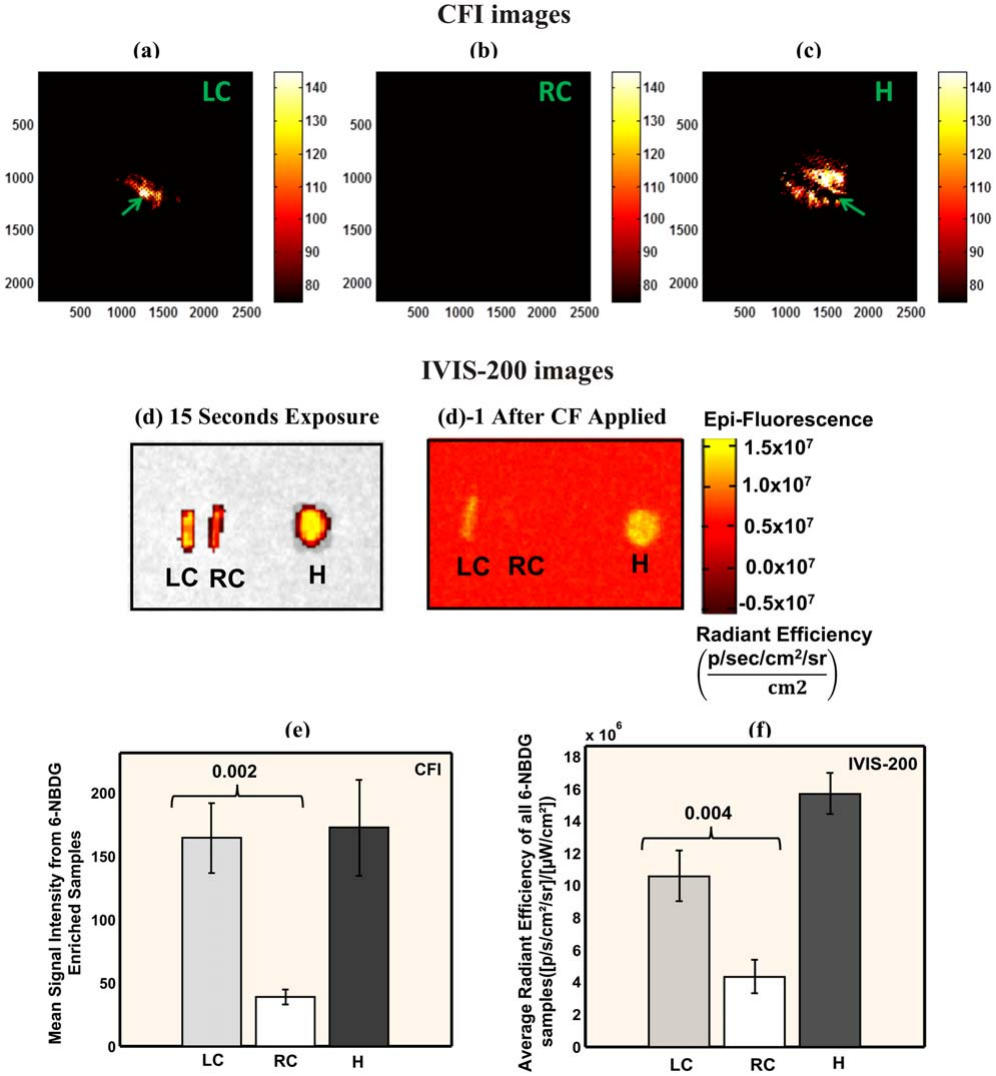
Fluorescence imaging of *ex vivo* murine carotids arteries. Fluorescence imaging was taken one hour after 6-NBDG intravenous injection (177 µL) with the CFI imaging system: (a–c) and with the IVIS-200 imaging system: (d, d-1). (a) LC: ligated left carotid artery with white area (green arrow) in the middle representing the ligation location, (b) RC: non-ligated right carotid artery artery (negative control), (c) H: heart (positive control). Dark line in the heart represents a gap in signal due the ventricular cavity (green arrow). (d) IVIS-200 images with GFP and GFP-background filters at 15 seconds exposure. (d)-1 processed image of (d) after a CF applied and background noise subtracted. (e) Average signal intensity of all samples (n = 3) was calculated based on the CFI system images. (f) Average radiant efficiency from the same samples is shown based on the IVIS-200 system images. High fluorescence signal from LCs was detected by the CFI system and confirmed by IVIS-200.

Fluorescence images of *ex vivo* plaques with the CFI system after 6-NBDG injection exhibited 4.3-fold greater fluorescence intensity (Figures 5a–c) from the ligated LCs than the non-ligated RCs (1.6×10^2^±2.7×10^1^ vs. 3.8×10^1^±5.9 A.U., P = 0.002; Figure 5e) with high signal from the heart. The LC is clearly visible with our system (Figure 5a) unlike the RC, which failed to emit sufficient fluorescence to overcome the sensitivity threshold of the CMOS camera. Thus, the RC provided an image (Figure 5b) that was similar to background (i.e., light source completely turned off). Our CFI system was also able to detect structural details of the LCs, such as location of the ligation (Figure 5a), which was not detectable through the IVIS-200 system. The IVIS-200 imaging system did confirm high signals from the LCs and heart, before and after CF applied (Figures 5d, 5d-1), which were 2.5–3.6× higher compared to the RCs (P = 0.004 for LC vs. RC, Figure 5f).

## Discussion

We have shown that our novel fiber-optic imaging system was sensitive enough to detect murine plaque uptake, *ex vivo*, of both ^18^F-FDG and 6-NBDG glucose probes. The clinically available ^18^F-FDG showed almost 5-fold higher signal intensity from murine carotid plaques compared to control, with 6-NBDG showing greater than 4-fold increase. Importantly, this is also the first demonstration of 6-NDBG as a glucose probe for atherosclerotic plaques.

Intravascular molecular imaging approaches utilizing optical imaging have demonstrated usefulness for biological imaging of plaques in coronary-sized vessels [31]. Based on this feasibility study we demonstrated that our dual-modality CRI-CFI prototype fiber-optic system was sufficiently sensitive towards the detection of ^18^F-FDG and 6-NBDG in atherosclerotic plaque with good resolution. Thus, our novel system in conjunction with radionuclide and fluorescence molecular probes may lead to intravascular imaging platforms for coronary arterial molecular imaging.

Coronary imaging remains challenging with PET or other currently available imaging modalities due to small size, constant motion, and in the case of ^18^F-FDG, obscuring uptake by adjacent myocardium [14]. Although ^18^F-FDG uptake is a marker for viable myocardium, this myocardial uptake makes it harder to image the adjacent small-sized coronaries [32], though there are some promising initial results [33]. This makes an intravascular imaging approach advantageous, minimizing the distance and maximizing the sensitivity to coronary plaque signal. While PET ^18^F-FDG has been well studied, there are other PET agents being investigated, such as ^18^F-labeld mannose [34], which could also be studied by our fiber-optic system.

Given the wealth of experimental and clinical data with ^18^F-FDG, identifying a fluorescent analog for *in vivo* intravascular imaging would be valuable. Intravascular fluorescence imaging [31], [35], [36] can use molecular and cellular fluorescent probes that are sensitive to metabolic processes of interest, such as inflammation, to obtain information about the underlying biology. 6-NBDG appeared to be the most promising fluorescent glucose marker based on prior studies in other disease models [17], [21], [37]. Our positive results with 6-NBDG, both in macrophage cell culture and murine plaques, show that further studies of this fluorescent glucose probe for atherosclerosis are warranted. This may complement other studies of fluorescent-based probes of enzymatic activity [15], [38].

The radionuclide imaging with the CRI system was designed to capture only β-particles. Although, we did not have any built-in γ-particle detector in our system, we performed experiments to confirm that no γ-particles interacted with the radioluminescence signal from the sample. Before imaging the LCs or RCs with the CRI system, we placed a slice of ^18^F-FDG enriched heart in close proximity (0.5 cm) and observed no background signal (Figure 3b). Even with the scintillator in such close proximity to γ-particles from the heart, we were still able to image the plaque location in the LCs without any background noise.

LSO (75% or 28,000 photons/MeV) was chosen as a scintillating material over BGO (15% or 8,200 photons/MeV) or plastic scintillator for its high relative light output or efficiency. Energy resolution that influences scatter rejection is also low for LSO (15–20%) compared to BGO (25%) and faster scintillating decay time (LSO: 40 nsec compared to BGO: 300 nsec). This allows fast coincidence timing with efficient rejection of random events to provide very high count rates [22]. As any LSO scintillating screen with a thickness as small as 1 mm provides an intrinsic efficiency close to 10% due to annihilation photons, we chose to use a scintillating screen with a thickness of 0.5 mm. Due to this extremely thin screen, the CRI system eliminates a significant amount of annihilation photons, which would have come from distant tissues and hinder the imaging of the local ^18^F-FDG uptake in the plaque. As this was a study to investigate the feasibility of the CRI system, we used a higher radiation dose than typically used. Future experiments will be needed to assess lower doses.

While we studied both ^18^F-FDG and 6-NBDG separately after intravenous delivery in an *ex vivo* atherosclerosis model, this study was limited as we did not perform *in vivo* intravascular imaging. Our system was built for dual-modality imaging to be advantageous for *in vivo* imaging, given that atherosclerosis can vary in extent and in location from subject to subject. The two modalities may also complement each other, for example in regards to signal depth and spatial localization. In the current study, 6-NBDG imaging appeared qualitatively to show more spatial detail, but this requires further study. *In vivo* animal studies comparing the two modalities will then inform whether a dual-modality system, or the superior single modality only, should be translated to future clinical imaging. The current manuscript demonstrates the feasibility of each component *ex vivo*, with further miniaturization/optimization needed to compare the modalities *in vivo*.

To miniaturize the system, an optimized wide-angle lens will be investigated, as well as further improvements in resolution/sensitivity (e.g., higher number of optical imaging fibers, lower temperature CCD camera) and flexible scintillating screen. The leached image bundle of the CRI needs to be modified to accommodate the delivery of excitation light for 6-NBDG, turning off the fluorescence light source during the radionuclide imaging.

While our study used a small number of animals, one of the advantages of this animal model is that the non-ligated contralateral carotid serves as a negative control, plus heart tissue serves as a positive control, so we imaged 3 specimens per animal. With this sample size, we were able to demonstrate statistical significance between ligated and non-ligated carotid signal for both ^18^F-FDG and 6-NBDG. A larger sample size will be needed for future *in vivo* studies comparing radionuclide and fluorescent agents.

## Conclusion

A dual-modality fiber-optic molecular imaging approach successfully detected both radionuclide (^18^F-FDG) and fluorescent (6-NBDG) glucose probes in murine atherosclerosis. This may provide new opportunities for investigating atherosclerosis and identifying high-risk coronary plaques.

## Supporting Information

Figure S1 **Fluorescence imaging of 6-NBDG uptake by macrophages.** (a) *In vitro* fluorescence imaging of RAW264.7 macrophage cells (10^5^ cells per well) with various concentrations of 6-NBDG fluorophore. (b) Significant positive correlation between the average radiant efficiency of all samples (n = 6) with exposure time 0.5–10 seconds (any exposure over 10 seconds saturated the *in vitro* fluorescence image taken with IVIS-200) and 6-NBDG concentrations. A quadratic relationship between signal and concentration was found to be the best fit and highly significant (p<0.0001 for all 3 exposure times).
(TIF)

Methods S1 (DOCX)

Results S1 (DOCX)
