# Supplementary figures and images for: Fiber-Optic System for Dual-Modality Imaging of Glucose Probes 18F-FDG and 6-NBDG in Atherosclerotic Plaques
Source: PLoS One. 2014 Sep 18;9(9):e108108. doi: 10.1371/journal.pone.0108108 (PMC4169475; doi:10.1371/journal.pone.0108108)

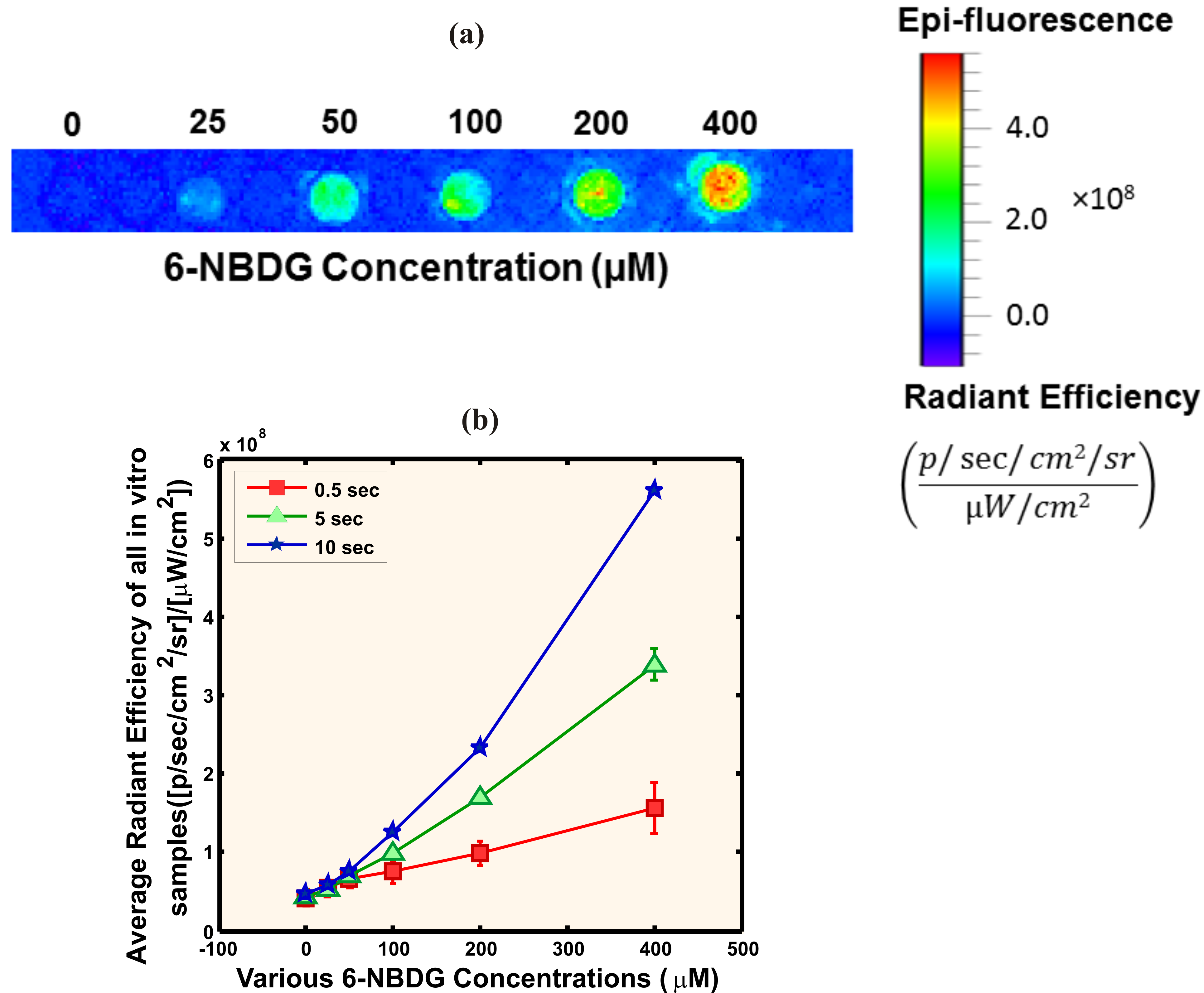

Supplement: Figure S1 — Fluorescence imaging of 6-NBDG uptake by macrophages. (a) In vitro fluorescence imaging of RAW264.7 macrophage cells (105 cells per well) with various concentrations of 6-NBDG fluorophore. (b) Significant positive correlation between the average radiant efficiency of all samples (n = 6) with exposure time 0.5–10 seconds (any exposure over 10 seconds saturated the in vitro fluorescence image taken with IVIS-200) and 6-NBDG concentrations. A quadratic relationship between signal and concentration was found to be the best fit and highly significant (p<0.0001 for all 3 exposure times). (TIF) [file pone.0108108.s001.tif]
