# Supplementary material for: Fiber-Optic System for Dual-Modality Imaging of Glucose Probes 18F-FDG and 6-NBDG in Atherosclerotic Plaques
Source: PLoS One. 2014 Sep 18;9(9):e108108. doi: 10.1371/journal.pone.0108108 (PMC4169475; doi:10.1371/journal.pone.0108108)
Supplement: Methods S1 — (DOCX) [file pone.0108108.s002.docx]

**SUPPLIMENTARY METHODS**

***In vitro* validation of 6-NBDG macrophage uptake**

To establish 6-NBDG fluorophore as a potential molecular marker for detecting macrophage-rich atherosclerotic plaque, we established macrophage uptake *in vitro.* We seeded mouse macrophage cells (RAW264.7, American Type Culture Collection, VA, USA) into 96-well plates (1x10^5^/ well). Three sets of cells were cultured for 24 hours. After washing with Phosphate buffered saline (PBS), cells were fasted in media without glucose for 1 hour, after which 6-NBDG was added to each well at multiple concentrations (0, 25, 50, 100, 200, 400 µM). One hour later, cells were washed with PBS and uptake of 6-NBDG was assessed with the IVIS-200 imaging system at multiple exposure times (0.5, 5, 10 seconds). The average radiant efficiency ([p/sec/cm^2^/sr]/[$\mu$W/cm^2^]) was calculated from the fluorescence image collected with the IVIS-200 system in a region of interest (ROI) after subtracting the background fluorescence signal and multiplying by a correction factor (CF). Any exposure time longer than 10 seconds saturated the camera.

**Statistical analysis of *in vitro* data**

For the *in vitro* study of 6-NBDG uptake, quadruplicate samples of macrophages were analyzed for average radiant efficiency at 6 different concentrations (0–400 µM) of 6-NBDG and 3 different exposure times (0.5–10 sec). Therefore, the data consisted of 72 independent observations, which were analyzed in an ANCOVA model based on a covariance matrix of average radiant efficiency as the outcome and both exposure time and concentration as fixed effects. The statistical analysis of the *in vitro* data was done in SAS software.
