# Supplementary material for: Fiber-Optic System for Dual-Modality Imaging of Glucose Probes 18F-FDG and 6-NBDG in Atherosclerotic Plaques
Source: PLoS One. 2014 Sep 18;9(9):e108108. doi: 10.1371/journal.pone.0108108 (PMC4169475; doi:10.1371/journal.pone.0108108)
Supplement: Results S1 — (DOCX) [file pone.0108108.s003.docx]

**SUPPLIMENTARY RESULTS**

Figure S1-a is an IVIS-200 image of macrophages at various concentrations of 6-NBDG (0–400 µM) for 10 seconds exposure time. The fluorescence signal showed a positive realtionship with increasing concentrations of 6-NBDG (Figure S1-b). Average radiant efficiencies varied between 4.6×10^7^±8.6×10^6^ and 5.6×10^8^±5.0×10^5^ [p/sec/cm^2^/sr]/[µW/cm^2^] for exposures between 0.5–10 seconds. A quadratic relationship between signal and concentration was found to be the best fit and highly significant (P<0.0001 for all 3 exposure times). In addition, the curves showed a significant increase in signal with longer exposure time (P<0.0001 for both 10 sec and 5 sec vs. 0.5 sec).
